# Structural Stability of Human Protein Tyrosine Phosphatase ρ Catalytic Domain: Effect of Point Mutations

**DOI:** 10.1371/journal.pone.0032555

**Published:** 2012-02-28

**Authors:** Alessandra Pasquo, Valerio Consalvi, Stefan Knapp, Ivan Alfano, Matteo Ardini, Simonetta Stefanini, Roberta Chiaraluce

**Affiliations:** 1 UT-BIORAD-FARM CR Casaccia ENEA, Rome, Italy; 2 Department of Biochemical Sciences “A. Rossi Fanelli”, Sapienza University of Rome, Rome, Italy; 3 Structural Genomics Consortium, Oxford University, Oxford, England, United Kingdom; National Institute for Medical Research - Medical Research Council, United Kingdom

## Abstract

Protein tyrosine phosphatase ρ (PTPρ) belongs to the classical receptor type IIB family of protein tyrosine phosphatase, the most frequently mutated tyrosine phosphatase in human cancer. There are evidences to suggest that PTPρ may act as a tumor suppressor gene and dysregulation of Tyr phosphorylation can be observed in diverse diseases, such as diabetes, immune deficiencies and cancer. PTPρ variants in the catalytic domain have been identified in cancer tissues. These natural variants are nonsynonymous single nucleotide polymorphisms, variations of a single nucleotide occurring in the coding region and leading to amino acid substitutions. In this study we investigated the effect of amino acid substitution on the structural stability and on the activity of the membrane-proximal catalytic domain of PTPρ. We expressed and purified as soluble recombinant proteins some of the mutants of the membrane-proximal catalytic domain of PTPρ identified in colorectal cancer and in the single nucleotide polymorphisms database. The mutants show a decreased thermal and thermodynamic stability and decreased activation energy relative to phosphatase activity, when compared to wild- type. All the variants show three-state equilibrium unfolding transitions similar to that of the wild- type, with the accumulation of a folding intermediate populated at ∼4.0 M urea.

## Introduction

The classical protein tyrosine phosphatase (PTP) superfamily includes 38 proteins which specifically dephosphorylate phosphotyrosine residues and, in concert with protein tyrosine kinases, control a large number of diverse biological processes, such as cell proliferation, adhesion, apoptosis and migration [1]–[6]. Reversible tyrosine phosphorylation controls numerous signaling pathways which require a right balance between kinase and phosphatase activity. The involvement of PTP in controlling cellular signaling has been largely recognized [2], [5], [7], though the role of PTP in human diseases has not been explored so extensively as that of protein kinases. However in diverse diseases, such as cancer, diabetes and immune deficiencies, dysregulation of Tyr phosphorylation has been observed [4], [7], [8].

On the basis of their counteracting activity on the oncogenic protein tyrosine kinase, PTPs have been initially considered as potential tumor suppressors, however it is clear that several phosphatases have oncogenic properties [2]–[6], [9]. Over the last decade a limited number of phophatases have been studied systematically to evaluate their role in tumorigenesis. In particular, six mutated phosphatases have been directly linked to colorectal cancers [1], [10]; among the six mutated genes, the *PTPRT* gene encoding PTPρ (PDB accession code 2OOQ) was found to be most frequently mutated and it was also mutated in about 20% of lung and gastric cancer [10].

PTPρ belongs to the classical receptor type IIB family of PTP. The 107 PTPs encoded by the human genome are classified into four classes, on the basis of the amino acid sequence of their catalytic domain. Class I includes 61 dual-specificity phosphatases and 38 classical phosphotyrosine-specific phosphatase which are further divided into receptor and non-transmembrane groups [5], [7]. The full-length PTPρ contains an extracellular domain, formed by a meprin-A5 antigen-PTP (MAM) domain and Ig-like and fibronectin type III-like repeats, a single transmembrane segment and one or two cytoplasmic catalytic domains. The catalytically active proximal D1 domain is adjacent to the membrane and is connected to the inactive membrane-distal D2 domain [3], [5]. The PTP membrane-proximal catalytic domain consists of about 280 residues that fold into a highly conserved α/β structure [4], [11]. Conserved functional elements of the catalytic PTP domain are the PTP signature motif, the mobile “WPD” loop, a highly conserved aspartate residue required for catalysis and the phosphotyrosine recognition loop.

In cancer tissues several PTPρ variants in the catalytic domain have been identified and there is evidence to suggest that PTPρ may act as a tumor suppressor gene [10]. These natural variants are nonsynonymous single nucleotide polymorphisms (nsSNPs), single nucleotide variations occurring in the coding region and leading to a polypeptide sequence with amino acid substitutions.

A large number of amino acid substitutions originate from nsSNPs and an increasingly large number of diseases and defects reported in Human Gene Mutation Database [12] and Online Mendelian Inheritance in Man (OMIM) [13] are referred to nsSNPs [14]. Although most nsSNPs are considered not to affect protein function, computational analysis predicts that around 30% of protein variants resulting from nsSNPs are less stable than the most common variant [15]. The effect of disease-causing nsSNPs on protein structure and function has been widely investigated by computational analysis, and change in protein stability has been suggested as the most common mechanism involved in monogenic disease [16]–[18]. However, nsSNPs may also affect and modulate the protein function by altering protein dynamics without affecting protein stability [19]. Notably, since genetic variations related to nsSNPs may influence individual susceptibility to complex diseases such as cancer [20] or response to drugs, a more extended study about the effect of nsSNPs on protein structure may help in understanding their role in inducing protein functional changes [21]. To date there are few experimental data available concerning the consequences of nsSNPs on protein function and stability.

In this study we investigate the effect of amino acid substitutions identified in colorectal cancer [10] and in the single nucleotide polymorphisms database [18], [22], [23] on the thermodynamic stability and on the activity of the membrane-proximal catalytic domain of PTPρ [1], [24]. The analysis revealed that, in comparison to the wild-type, the thermal and thermodynamic stability of all the mutants are decreased as well as the activation energy relative to the phosphatase activity, indicating an increase in protein flexibility of all the PTPρ mutants. All the variants show three-state equilibrium unfolding transitions similar to that of the wild-type, with the accumulation of a folding intermediate at ∼4.0 M urea.

To our knowledge, this study represents the first spectroscopic and thermodynamic characterization of human PTPρ catalytic domain and some of its mutants found in cancer. In addition, the effects of none of the mutations reported in this manuscript on protein thermodynamic stability has been previously investigated.

## Results

**Figure 1:**
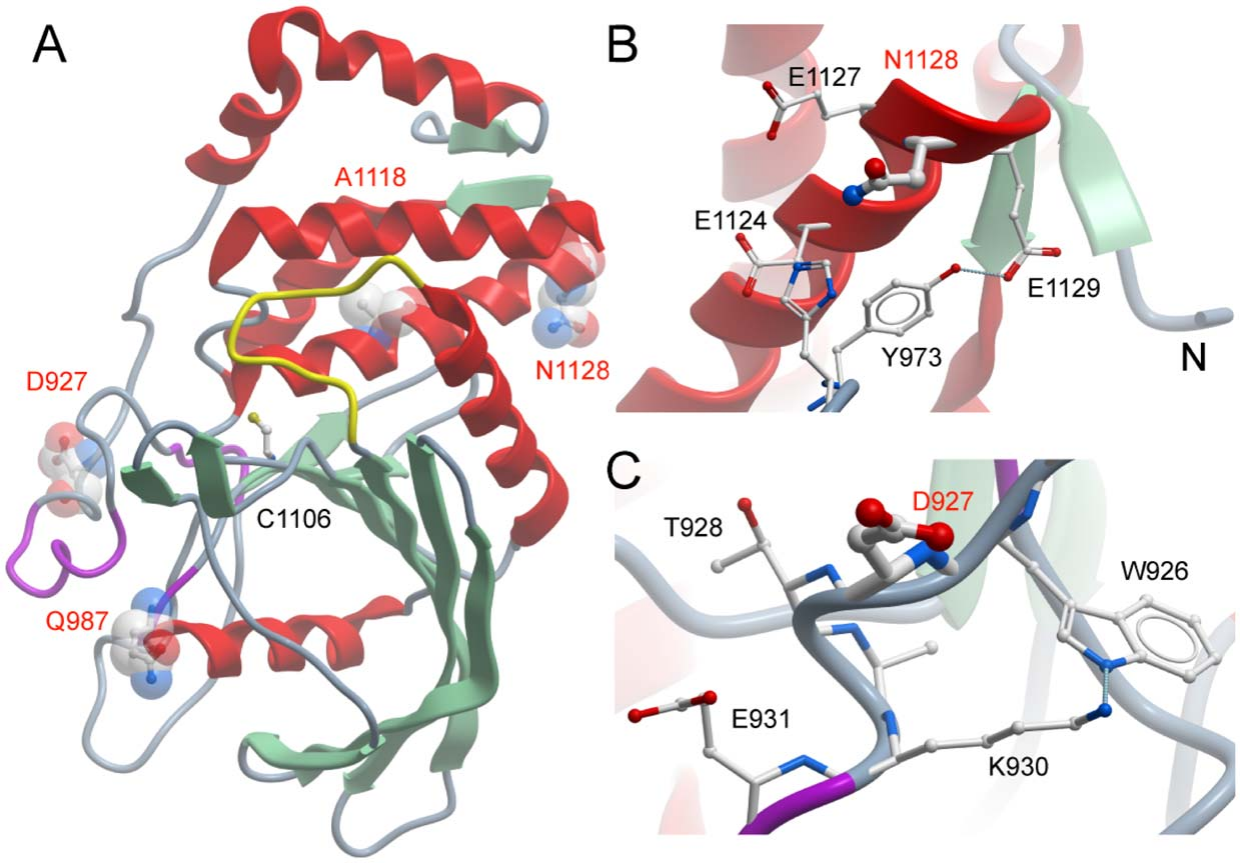
Location of PTPρ mutations. (**A**) Structure of PTPρ (PDB code 2OOQ) shown as a ribbon diagram. Mutated residues are highlighted in ball and stick and transparent cpk representation and are labelled in red. The active site cystein residue (C1106) is also shown and the catalytically important WPD loop is coloured in yellow. (**B**) Local environment of N1128. (**C**) Local environment of D927.

Four mutations of the PTPρ membrane-proximal catalytic domain in public databases have been identified: D927G, Q987K, N1128I and A1118P. Introduction of these mutations resulted in soluble recombinant protein only for D927G, Q987K and N1128I whereas the A1118P mutant could not be expressed in the soluble fraction even when different growth and induction conditions were used. Mapping of the mutations onto the structure of the PTPρ catalytic domain revealed that A1118P is located in the middle of a central helix (Fig. 1). It is likely that the introduction of a proline residue at this position in substitution of alanine will break the helical secondary structure resulting in misfolding of the catalytic domain. N1128 is located at the C-terminal end of the same helix (Fig. 1B). This residue is located in a very polar environment with flanking acidic (E1124, E1127, E1129) and a histidine residue (H974) located in a neighbouring loop region. D927G and Q987K are located in solvent exposed loops with little interaction with other amino acid side chains (Fig. 1C). The solvent accessibility of the mutated residues Asp-927 and Gln-987 is more than 70% and Asn-1128 is 49% solvent exposed. The mutation D927G involves a residue (Asp-927) placed in a 4 residues turn between two coils. In the wild-type Asp-927 connects different protein secondary structure regions through hydrogen bonds with three residues. One hydrogen bond is between the N of the peptide bond and the OD2 of Asp-947 and the other two hydrogen bonds are between the carbonyl of the peptide bond and the amidic nitrogens of Lys-930 and Glu-931. The carboxylic moiety of Asp-927 is completely solvent exposed and does not apparently make any contact with other residues.

### Spectroscopic characterization of PTPρ wild-type and its mutants

**Figure 2:**
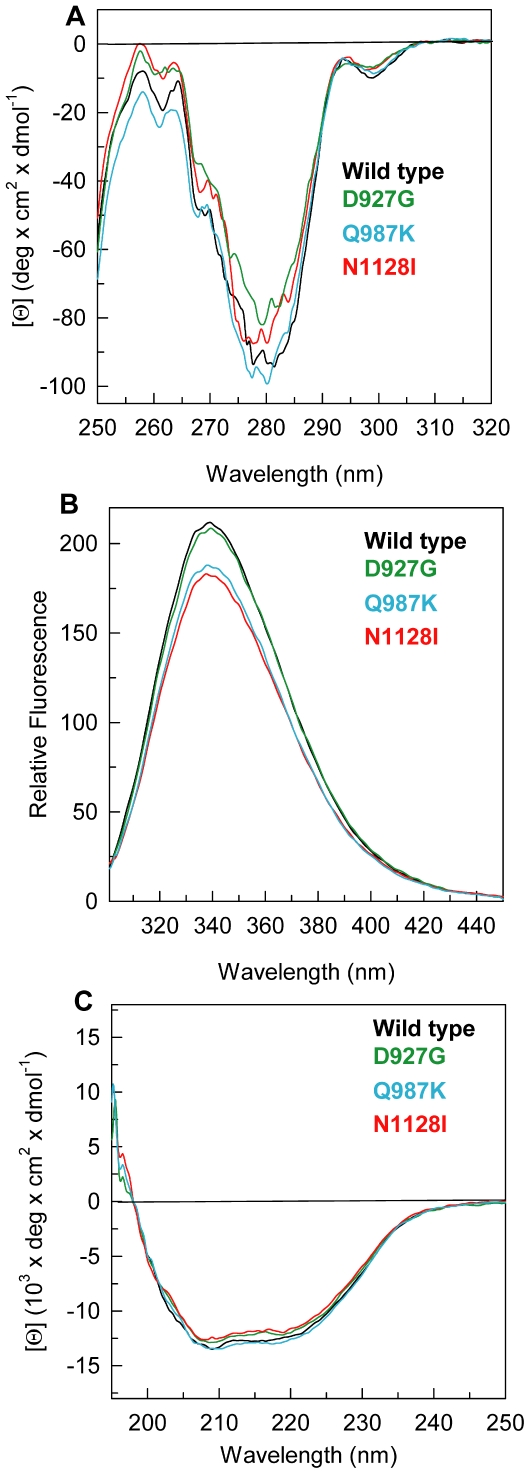
Spectroscopic properties of PTPρ wild-type and mutants. (**A**) Near-UV CD spectra were recorded in a 1-cm quartz cuvette at 1.0 mg/ml protein concentration in 20 mM Tris/HCl, pH 7.5 containing 0.2 M NaCl and 2 mM DTT. (**B**) Intrinsic fluorescence emission spectra were recorded at 0.04 mg/ml protein concentration (295 nm excitation wavelength) in 20 mM Tris/HCl, pH 7.5 containing 0.2 M NaCl and 200 µM DTT. (**C**) Far-UV CD spectra were recorded in a 0.1-cm quartz cuvette at 0.2 mg/ml in 20 mM Tris/HCl, pH 7.5 containing 0.2 M NaCl and 0.4 mM DTT.

The near-UV CD spectrum of wild-type PTPρ shows the contribution of all aromatic residues and is characterized by two negative components centred at 280 nm and at around 298 nm accompanied by fine structure features at 275–280 nm (Fig. 2A). Q987K variant displays a near-UV CD spectrum almost identical to that of the wild-type as well as D927G and N1128I, which nevertheless show a slight decrease of the dichroic activity at 275–280 nm. In line with the near-UV CD results, which suggest a similar tertiary arrangement for the wild-type and all the variants, the fluorescence emission spectra of mutants are similar to that of wild-type protein, being all centred at the same maximum emission wavelength around 338 nm but characterized by a decreased emission fluorescence intensity (Fig. 2B). Far-UV CD spectra of all the PTPρ mutants superimpose well with that of the wild-type and are typical of an alpha and beta protein, showing a local minimum at around 208 nm, a 200 nm zero intercept and a 1.13 ratio of the 208/222 bands (Fig. 2C). These results indicate that the SNP mutations had no effect on the secondary structure of the protein and suggest that, in the native state, the effect of the mutations are directed and localized to the mutated residue with minor modification of tertiary arrangements.

### Thermal unfolding

**Figure 3:**
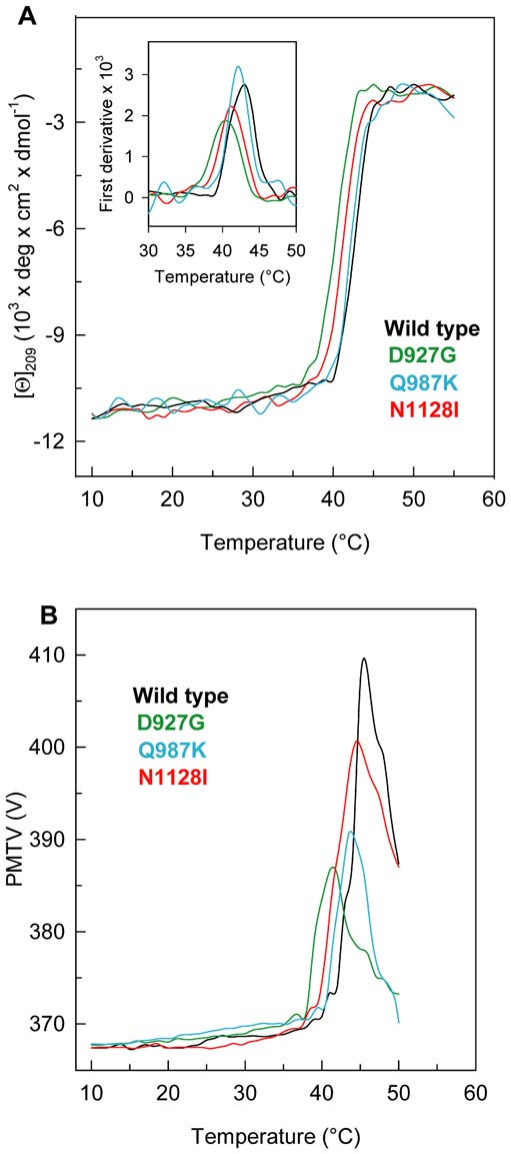
Thermal transition of PTPρ wild-type and mutants. (**A**) PTPρ wild-type, N1128I, Q987K and D927G were heated from 10°C to 72°C in a 0.1-cm quartz cuvette at 0.2 mg/ml in 20 mM Tris/HCl, pH 7.5 containing 0.2 M NaCl and 0.4 mM DTT. The dichroic activity at 209 nm was monitored continuously every 0.5°C. The inset shows the first derivative of the same data. (**B**) PMTV data recorded in the same experiments shown in (**A**).

The thermal stability of D927G, Q987K and N1128I was investigated by continuously monitoring the ellipticity changes at 209 nm between 10 and 72°C in comparison with that of wild-type (Fig. 3). The parameter chosen to compare the transition curves of PTPρ wild-type and mutants is the melting temperature (T_m_) defined as the mid point of the denaturation process calculated by plotting the first derivative of the molar ellipticity values as a function of temperature (Fig. 3, inset). The temperature-induced ellipticity changes at 209 nm, where the main amplitude was observed, occur in an apparent cooperative transition for PTPρ wild-type, Q987K, N1128I and D927G, and with apparent T_m_ values of 43.0, 42.0, 41.0 and 40.0°C, respectively. The temperature-induced ellipticity changes for wild type and mutants are all coincident with the heat-induced increase of the photomultiplier tube voltage (PMTV) above 370 V (Fig. 3B), suggesting that the temperature-induced unfolding is accompanied by protein aggregation [25]. Aggregation occurred also when thermal scans were performed at a lower heating rate with a low-temperature shifts of the apparent T_m_; the differences between the apparent T_m_ of wild type and variants were the same as those measured at higher heating rate (data not shown). The observed transitions are irreversible as indicated by the spectra measured at the end of the cooling phase that differ from those of the native proteins measured at the beginning of the thermal transitions (data not shown). Furthermore, inspection of the cuvette at the end of the cooling phase revealed the presence of a large amount of precipitate in all the samples.

### Temperature dependence of phosphatase activity

**Figure 4:**
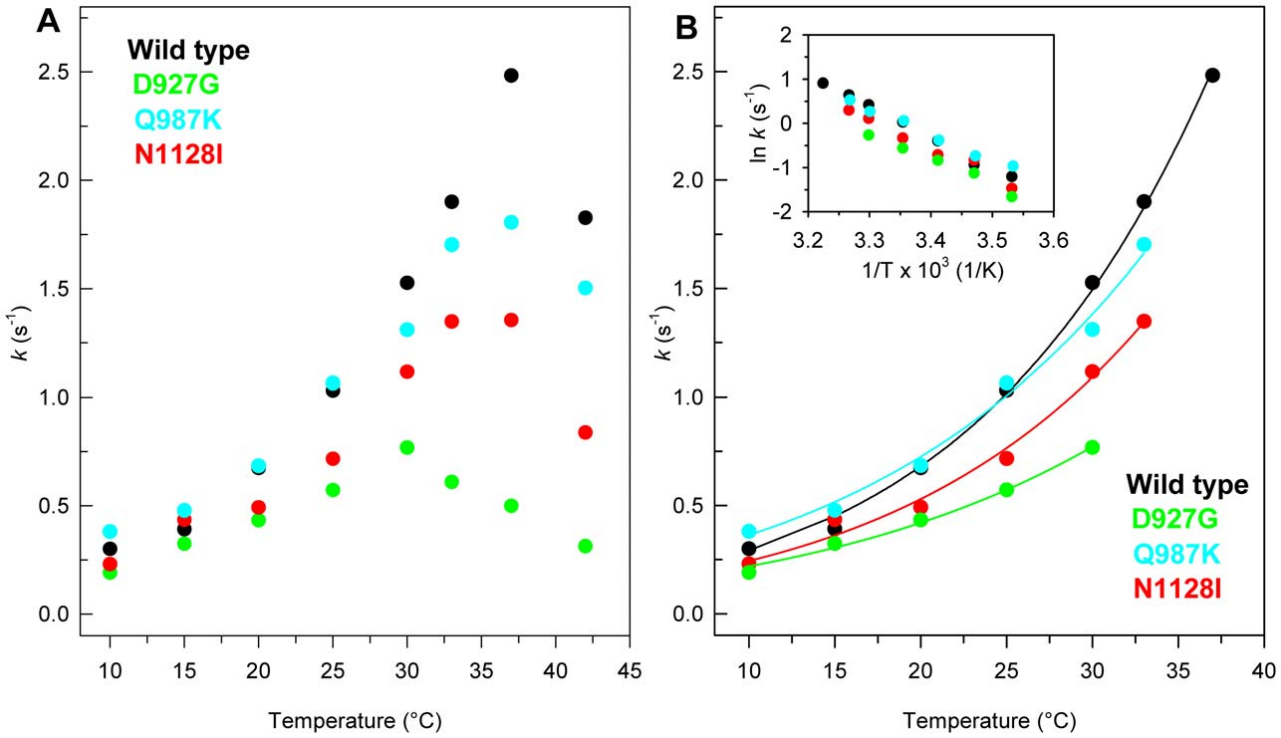
Effect of temperature on phosphatase activity of PTPρ wild-type and mutants. (**A**) Temperature dependence of phosphatase activity of PTPρ wild-type and mutants. (**B**) Non-linear fit of the temperature dependence of phosphatase activity to the Arrhenius equation (Eqn. 1); the inset shows the linear Arrhenius plot for the same data. Assays were performed under the conditions described in Materials and Methods, using 1 nM enzyme.

The temperature dependence of phosphatase activity of PTPρ wild-type and variants was examined over the temperature range of 10–42°C (Fig. 4A). The optimal temperatures were estimated to be at 37°C for wild type and Q987K, at around 33°C for N1128I and at 30°C for D927G (Fig. 4A). Notably, at 37°C, the phosphatase activity of all PTPρ variants is significantly reduced and corresponds to 72, 54 and 20% of that of the wild-type protein for Q987K, N1128I and D927G, respectively. The activation energy, *E*_a_^‡^, determined by the Arrhenius equation (1) in the temperature range between 10°C and the optimal temperature of each protein, corresponds to 13.88±0.40 kcal/mol for the wild-type and to 12.77±0.51, 10.73±0.54 and 11.38±0.46 kcal/mol for N1128I, D927G and Q987K (Fig. 4B), respectively. This result suggests an increased flexibility of all the variants compared to the wild-type, particularly evident for N1128I and D927G whose phosphatase activity is significantly decreased at 42°C. The comparison of temperature dependence of phosphatase activity with the structural thermal unfolding monitored at 209 nm clearly indicates that the D927G and N1128I variants are significantly more flexible and less thermal resistant than the wild-type.

### Urea-induced equilibrium unfolding transitions

PTPρ wild-type and variants reversibly unfold in urea at 10°C in 20 mM TrisHCl, pH 7.5, containing 200 µM DTT and 0.2 M NaCl. The effect of increasing urea concentrations (0–8 M) on the structure of PTPρ mutants was analyzed by far-UV CD and intrinsic fluorescence emission spectroscopy and compared to the wild-type.

**Figure 5:**
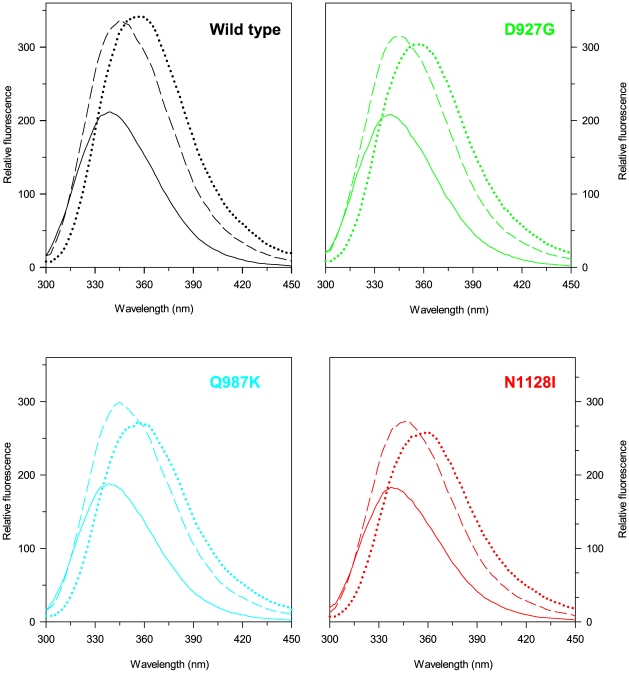
Intrinsic fluorescence emission spectra of PTPρ wild-type and mutants. Fluorescence spectra of PTPρ wild-type and mutants in 0 M (continuous lines), 8.30 M (dotted lines), 3.95 M (D927G and N1128I, dashed lines) and 4.45 M urea (wild-type and Q987K, dashed lines) were recorded at 0.04 mg/ml protein concentration (295 nm excitation wavelength) at 10°C in 20 mM Tris/HCl, pH 7.5 containing 0.2 M NaCl and 200 µM DTT.

**Figure 6:**
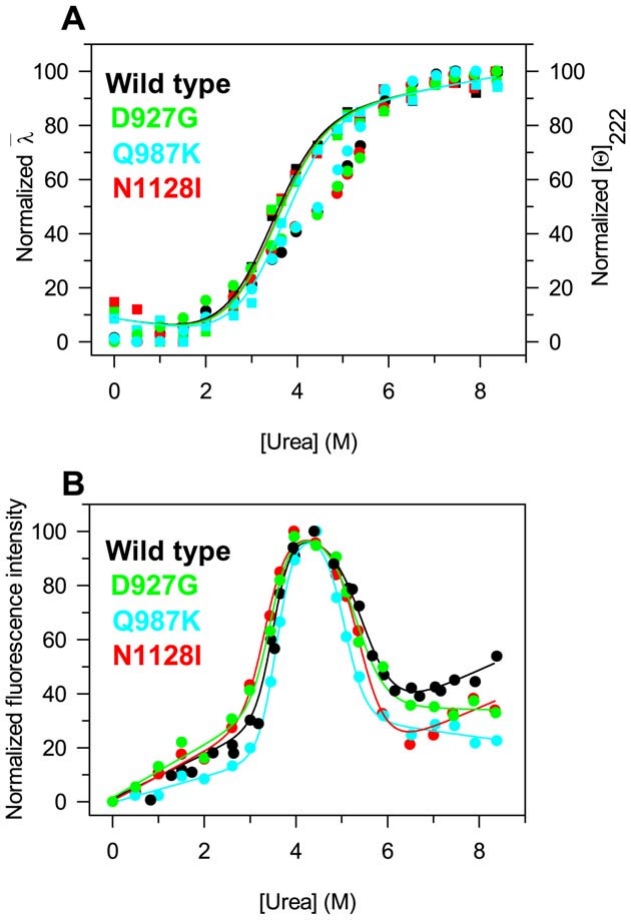
Urea-induced equilibrium unfolding of PTPρ wild-type and mutants. (**A**) Normalized intensity-averaged emission wavelength (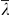, left axis, filled circles) and molar ellipticity at 222 nm reported after removal of the high-frequency noise and the low-frequency random error by SVD ([Θ]_222_, right axis, empty squares); the continuous lines represent the nonlinear global fitting of the molar ellipticities at 222 nm data to Eqn. 4 for PTPρ calculated as described in Materials and Methods. (**B**) Normalized relative fluorescence intensities at 338 nm; the continuous lines represent the nonlinear regression fit of the relative fluorescence intensities at 338 nm to Eqn. 6 calculated as described in Materials and Methods. The reversibility points (empty circles) are shown, for clarity, only for the relative fluorescence intensities of the wild-type and were not included in the nonlinear regression analysis. All spectra were recorded at 10°C as described in Materials and Methods.

Incubation of PTPρ wild-type and variants at increasing urea concentrations at 10°C for 30 min, a time sufficient to reach the equilibrium, resulted in a progressive change of the intrinsic fluorescence emission intensity and a red-shift of the maximal emission wavelength. At the end of the transition, above 7 M urea, the intrinsic fluorescence emission intensity is increased about 1.5 fold and the maximal fluorescence emission wavelength shifts to around 358 nm either for the wild-type and all the variants (Fig. 5). Determination of the red-shift of the intrinsic fluorescence emission was obtained by calculating the intensity averaged emission wavelength 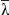 at increasing urea concentration (Fig. 6A). This parameter is an integral measurement, negligibly influenced by the noise, and reflects changes in both the shape and the position of the emission spectrum.

The same samples used to monitor the fluorescence emission changes during the unfolding transition were used to monitor far-UV CD ellipticity (Fig. 6A). The unfolding process is fully reversible upon dilution of the denaturant, either for the wild-type as well as for all the mutants (Fig. 6A). The urea-induced changes in intensity averaged emission wavelength 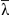 and in 222 nm ellipticity of all the mutants are similar to that of the wild-type and show a sigmoidal dependence on urea concentration, following an apparent two-state transition. However, the transitions monitored by ellipticity changes and 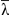 changes do not coincide (Fig. 6A) suggesting the possible presence of an intermediate in the transition region at approximately 4 M urea for the wild-type and for all the variants. The plot of the relative fluorescence intensity changes versus urea concentration shows a complex dependence upon increasing denaturant concentration for the wild-type and all the mutants (Fig. 6B). The data in Fig. 6B clearly indicate a non two-state unfolding process and the population of a denaturation intermediate at about the same urea concentration of the apparent denaturation midpoints observed by ellipticity and 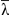 averaged changes (Fig. 6 A and B). The folding intermediate detected by relative fluorescence intensity is populated at around 4.45 M urea for wild-type and Q987K and at around 3.95 M urea for D927G and N1128I. The intrinsic fluorescence emission spectra of the folding intermediate are similar for all the proteins: the fluorescence intensity is increased of about 1.5 fold and the maximum emission wavelength is shifted from 338 to 345 nm, with respect to the native state (Fig. 5). The hyperfluorescent intermediate of wild-type and variants retains about 35% of the 222 nm ellipticity of the native state (Fig. 6A).

The transitions monitored by relative fluorescence intensity changes (Fig. 6B) were fitted to a three-state unfolding process which yielded the thermodynamic parameters for wild-type and the variants of PTPρ (Table 1). For the first transition, *m* values are all closely similar to that of the wild-type, suggesting a similar unfolding mechanism for all the variants; Δ*G* values lower than that of the wild-type are observed for N1128I and D927G, suggesting a destabilization of the native state for these two variants, that also show a decreased thermal stability (see Fig. 3 and 4). For the second transition, that represents the unfolding of the intermediate to the denatured state, the *m* values of all the proteins are lower than those of the first transition, indicating that a larger increase in solvent-exposed surface area occur in the unfolding from the native to the intermediate state (Table 1). Notably, Δ*G* values relative to the second unfolding transition for N1128I and Q987K are larger than those relative to the first transition, suggesting a higher stability of the intermediate states, compared to that of the wild-type.

**Table 1:** Thermodynamic parameters for urea-induced unfolding equilibrium of PTPρwild-type and mutants.

	Wild-type	N1128I	D927G	Q987K
*m*_I-N_ (kcal/mol/M)	2.94±0.32	2.43±0.28	2.69±0.45	2.98±0.27
D50_I-N_ (M)	3.51±0.02	3.34±0.03	3.46±0.03	3.58±0.02
Δ*G* ^H^_2_^O^_I-N_ (kcal/mol)	10.32	8.12	9.31	10.67
*m*_U-I_ (kcal/mol/M)	1.61±0.13	1.91±0.18	1.81±0.26	2.51±0.25
D50_U-I_(M)	5.49±0.03	5.40±0.03	5.31±0.05	5.06±0.02
Δ*G* ^H^_2_^O^_U-I_ (kcal/mol)	8.84	10.31	9.61	12.70

Urea-induced unfolding equilibrium data were obtained at 10°C in 20 mM Tris/HCl, pH 7.5, containing 0.2 M NaCl and 200 µM DTT by measuring the relative fluorescence intensity at 338 nm. The free energy of unfolding from the native state to the intermediate (Δ*G* ^H^_2_^O^_I-N_) and from the intermediate to the unfolded state (Δ*G* ^H^_2_^O^_U-I_) were calculated from Eqn. 5. *D*50_I-N_ and *m*_I-N_ which are the midpoint and *m* value for the transition between native and intermediate state, respectively, and *D*50_U-I_ and *m*_U-I_ are the midpoint and *m* value for the transition between the intermediate and the unfolded state, respectively, were calculated from Eqn.6. Data are reported ± SE of the fit.

**Figure 7:**
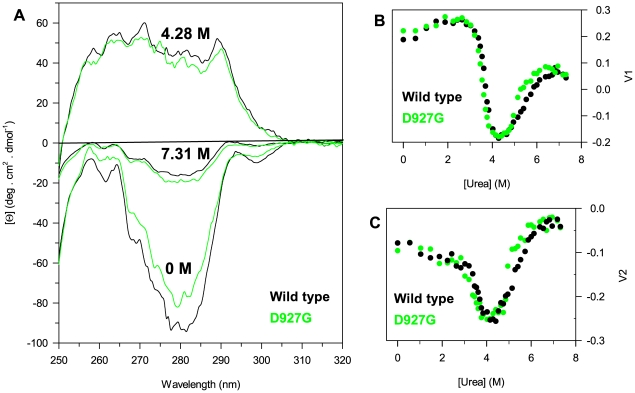
Effect of urea on near-UV CD spectra of PTPρ wild-type and D927G. (**A**) Near-UV CD spectra of wild-type and D927G in 0 M, 4.28 M and 7.31 M urea were recorded in a 1-cm quartz cuvette at 2.4 mg/ml protein concentration at 10°C in 20 mM Tris/HCl, pH 7.5 containing 0.2 M NaCl and 2 mM DTT. (**B**) and (**C**) Near-UV CD changes of wild-type and D927G at increasing urea concentrations reported as the first (V1, **B**) and the second (V2, **C**) column of the V matrix. V1 and V2 were obtained by SVD of the near-UV CD spectral data as described in the text.

The near-UV CD changes of D927G, the most temperature sensitive variant, upon increasing urea concentrations were monitored in comparison with wild-type (Fig. 7) to better characterize the nature of the intermediate state. The resulting data were analyzed after removal of the high-frequency noise and the low-frequency random error by a singular value decomposition algorithm (SVD) which resolved two main spectra components either for wild-type or for the mutant D927G. The most significant singular values were 2.05×10^3^ for wild-type and 1.96×10^3^ for D927G, the second singular values were 38.2% and 40.1% of the first singular value for wild-type and D927G, respectively. The plots of the first (*V*1) and of the second column (*V*2) of the **V** matrix which reflect the global change in the 250–320 nm region as a function of urea concentration show non-two state transition profiles comparable to the transitions monitored by intrinsic fluorescence emission intensity and confirm the accumulation of an intermediate at around 4.2 M urea (Fig. 7 B, and C). At this denaturant concentration, the near-UV CD spectra of wild-type and D927G significantly differ from those of the native state and are completely opposite in sign (Fig. 7A). The 298 nm negative band is lost and a positive contribution at 289 nm is present in the spectra of the intermediate state of both proteins. Furthermore, the 280–260 nm region, dominated by contribution of Phe and Tyr residues, in D927G is less defined than the wild-type (Fig. 7A). The data clearly indicate that both the spectral components of the wild-type and D927G contribute to the urea induced transitions with the accumulation of a denaturation intermediate at the same urea concentration range observed by monitoring intrinsic fluorescence intensity (Fig. 5 and Fig. 6B).

The urea-induced unfolding transitions monitored by far-UV CD ellipticity changes are non-coincident with those monitored by fluorescence (Fig. 6) and near-UV CD (Fig. 7). To identify the number of spectral components contributing to the urea-induced ellipticity changes, far-UV CD spectra were analysed after removal of the high-frequency noise and the low-frequency random error by SVD. The global changes in the spectral region 213–250 nm, analyzed by SVD, indicate that only two spectral components contribute to the far-UV CD spectra of wild-type and all the variants. The most significant singular values are very similar for wild-type and all the variants and range between 1.76×10^5^ and 1.67×10^5^. The second singular values are 19.8% of the first singular value for the wild-type, 16.6% for the D927G variant, 11.2% for Q987K and 8.0% for N1128I. All the other singular values are below 7.0% of the largest singular value and progressively decrease approaching to zero. The plots of the first column of the **V** matrix (*V*1), which reflect the global change in the 213–250 nm region, as a function of urea concentration (data not shown) show sigmoidal transition profiles comparable to the transitions monitored at 222 nm for the wild-type and the variants (data not shown). Non cooperative changes of *V*2 at increasing denaturant are observed. Furthermore, the reconstituted spectra of the first spectral component superimposed well with those of the sum of the first and the second spectral component (data not shown), indicating that only the first spectral component contributed to the urea induced transitions monitored by ellipticity at 222 nm. These results indicate an apparent two-state process for far-UV CD unfolding transitions of wild-type and variants. Thus the data have been globally fitted to a two-state model according to equation 4 with the *m* parameter shared between all the data sets. The free energy of unfolding, Δ*G* ^H^_2_^O^, of wild-type and mutants obtained from the global fitting are very similar and correspond to 3.56±0.09, 3.63±0.10, 3.65±0.10 and 3.82±0.07 kcal/mol for wild-type, N1128I, D927G and Q987 K, respectively, for a shared *m* value of 1.06±0.05 kcal/mol/M. Equilibrium unfolding of PTPρ wild-type and variants occurs *via* a hyperfluorescent intermediate state that cannot be detected by far-UV CD. Hence, the thermodynamic folding intermediate may represent a conformational change which occurs in the proximity of any of the tryptophans most likely due to an increase of native state flexibility [26].

## Discussion

PTPρ, which belongs to the classical receptor type IIB family of PTP, is one of the most frequently mutated PTP in human cancers [1], [24]. In this study we investigated the effect of amino acid substitution on the thermal and thermodynamic stability and on the activity, of the membrane-proximal catalytic D1 domain of PTPρ. Catalytically active fragments of several PTPs of type IIB family are proteolytically cleaved and released within the cytoplasm of tumour cells [27]–[29], hence the biophysical characterization of this catalytic domain may be relevant. The D1 domain is a monomeric, structurally independent and stable folding unit characterized by a high thermodynamic stability. In this study, we selected PTPρ mutations of D1 domain found in colorectal cancers [10] and reported in nsSNP database [14], D927G, Q987K, A1118P and N1128I. All the mutants are expressed as soluble recombinant proteins, except A1118P which remains insoluble also upon different growth and induction conditions. The spectroscopic properties of the wild-type compared with those of all the variants indicate that the mutations D927G, Q987K and N1128I had no effect on protein secondary structure with minor modifications of tertiary arrangements.

All the mutants show a decrease in the thermal stability and in the activation energy for phosphatase activity with respect to the wild-type indicating an increase in protein flexibility of all the mutants. Hence, at 37°C the phosphatase activity of all the variants was significantly reduced, similarly to what reported by Wang et al. for Q987K and N1128I [10]. The most destabilizing mutation, D927G, yielded a protein that at physiological temperature was almost completely inactive.

All the mutations studied here are located at very diverse positions in the structure, distant from the catalytic site and, except A1118P, in solvent-exposed loop regions. Presumably, the change in main chain flexibility caused by the most destabilizing D927G mutation may lead to local disorder and may affect the stabilizing hydrogen bonds of residues in close proximity (Asp-947, Lys-930 and Glu-931). This hypothesis is in agreement with the changes in local environment of aromatic residues suggested by the comparison of the near-UV CD spectrum of D927G with that of the wild-type. The N1128I mutation is less destabilizing than D927G. In the wild-type structure N1128, about 50% solvent accessible, is located in a very polar environment at the C-terminus of helix 8 and is involved, through its peptidic nitrogen, in a hydrogen bond with the peptide carbonyl of Asp-1124. The susbtitution of a polar residue, Asn-1128, to a nonpolar residue, Ile, may also contribute to destabilize the N1128I mutant. Q987K is the least destabilizing mutation with regard to thermal stability and temperature dependence of phosphatase activity, probably due to the fact that Gln-987 in the wild-type structure is not involved in any interaction with other protein residues.

All the variants, as revealed by intrinsic fluorescence emission intensity, show three-state equilibrium unfolding transitions similar to that of the wild-type, with the accumulation of a hyperfluorescent intermediate at ∼4.0 M urea that retains 35% of the native secondary structure ellipticity. The hyperfluorescent intermediate of wild-type and of D927G, the most temperature sensitive variant, contains tertiary contacts, although the packing of aromatic side-chains appears different from that of the native state.

The reversibility of the urea-induced unfolding equilibrium at low temperature allows a quantitative determination of the effect of mutations on the thermodynamic parameters of PTPρ catalytic domain. The destabilizing effect of mutations caused by nsSNPs on PTPρ thermodynamic stability (Table 1) may be referred i) to a destabilization of the native state for N1128I and D927G, as indicated by the values of Δ*G* ^H^_2_^O^_I-N_ lower than that of the wild-type, and ii) to a stabilization of the intermediate for all the variants, as suggested by the higher free energy of unfolding from the intermediate to the unfolded state (Δ*G* ^H^_2_^O^_U-I_), with respect to that of the wild-type (Table 1). The destabilizing effect of D927G and N1128I substitutions is also evident from the decrease in melting temperature monitored by secondary structure changes and from the significant reduction of phosphatase activity at 37°C that suggests a higher flexibility of the two variants with respect to the wild-type.

The reversible equilibrium unfolding transitions monitored by far-UV CD are not coincident with those monitored by near-UV CD and intrinsic fluorescence emission intensity and do not reveal any denaturation intermediate. The thermodynamic parameters relative to the apparent two-state equilibrium unfolding measured by far-UV CD do not indicate any significant difference between the variants and the wild-type and are lower than those determined by intrinsic fluorescence emission intensity. In particular, the shared *m* value of 1.06 kcal/mol/M calculated from global fitting of the far-UV CD equilibrium unfolding data is significantly lower than the predicted *m* value for a monomeric protein of 280 amino acid residues unfolded in urea which is 3.71±0.3 kcal/mol/M [30], a value closely similar to that measured by fluorescence intensity. As a matter of fact, fluorescence intensity is extremely sensitive to the microenvironment of a fluorophore and is considered as the most straightforward signal that can be related to thermodynamics of unfolding transitions [31]. The discrepancy between the thermodynamic parameters obtained by far-UV CD and fluorescence intensity and the lack of a detectable intermediate by far-UV CD may indicate that the hyperfluorescent intermediate state represents conformational changes which occur in the proximity of any of the tryptophans, with an alternative tertiary arrangement. In the native state, the maximum emission wavelength (λmax = 338 nm) indicate that tryptophan residues are shielded from the solvent, whereas a partial exposure of tryptophan residues to the solvent is observed for the intermediate (λmax = 345 nm). These results are in agreement with the structural data which indicate that in PTPρ two out of the five tryptophans, Trp-994 and Trp-998, are located in the same helical region (helix 6) and Trp-926, Trp-1023 and Trp-1072 are placed in coil regions. All the five tryptophans are almost completely buried. In the native state the solvent accessibility of the five chromophores is 15% for Trp-998, 14% for Trp-926 and 4% for Trp-1023, whereas all the other tryptophan residues are completely buried. Hence, at approximately 4.0 M urea, it is impossible to assign to any of the five chromophores a particular role in the fluorescence properties of the intermediate state.

In conclusion our results revealed a destabilizing and inactivating role of the mutations of PTPρ D1 domain found in colorectal cancers [10]. All the amino acid mutations studied here are on the surface of PTPρ and could potentially participate in protein-protein interactions. The stabilization of a folding intermediate, coincident with an alternative tertiary arrangement, may play a role in recognition and interaction with other substrate proteins as well as in sensitivity of this phosphatase to degradation.

## Materials and Methods

### Site-directed mutagenesis

PTPρ wild-type enzyme plasmid was obtained by SGC (Oxford) [8]. Quick Change Site-Directed Mutagenesis Kit (Stratagene) was used to introduce the single mutations on wild-type PTPρ plasmid used as template. The mutagenic oligonucleotides used are listed in Table 2.

**Table 2:** Primers sequences for mutagenesis of PTPρ.

Mutant	Primer sequences (5′ to 3′)
**Q987K**	Forward: GCGACTCAAGGTCCGATGAAGGAGACTGTAAAGGAC
	Reverse: GTCCTTTACAGTCTCCTTCATCGGACCTTGAGTCGC
**A1118P**	Forward:CGGACTGGCTGCTTCATTCCCATTGACACCATGCTTGACATGGC
	Reverse:GCCATGTCAAGCATGGTGTCAATGGGAATGAAGCAGCCAGTCCG
**N1128I**	Forward: GCTTGACATGGCCGAGATTGAAGGGGTGGTGG
	Reverse: CCACCACCCCTTCAATCTCGGCCATGTCAAGC
**D927G**	Forward: GCAGACAGCTTCGTGGGGCACAGCCAAGGAGG
	Reverse: CCTCCTTGGCTGTGCCCCACGAAGCTGTCTGC

Mutations were introduced using a Quick Change Site-Directed Mutagenesis Kit (Stratagene) with the listed primers.

### Protein Expression and Purification

PTPρ wild-type and mutants were expressed in *E. coli* strain BL21(DE3). 10 ml of overnight culture was grown at 37°C in 1 L LB media containing kanamycin as antibiotic at a final concentration of 50 µg/ml until optical density OD_600_ reached 0.6. The culture was cooled on ice for 20 min, then the protein expression was induced overnight by adding 0.5 mM isopropyl-β-D-thiogalactoside (Sigma-Aldrich) and grown overnight at 15°C with energic shaking. The culture was harvested by centrifugation and resuspended in 50 ml of Binding buffer (50 mM Hepes, 500 mM NaCl, 5 mM Imidazole, 5% Glycerol, pH 7.5) containing 0.5 mM *tris*(2-carboxyethyl)phosphine, and stored at −20°C until use. The cells were thawed on ice supplemented with protease inhibitors (Complete, Roche) and disrupted by sonication. The lysate was cleared by centrifugation and the supernatant was loaded on a DE52 column (GE Healthcare), previously equilibrated with Binding buffer and 0.5 mM *tris*(2-carboxyethyl)phosphine, to remove nucleic acids. The flow-through was loaded on a Ni-NTA (Ni^2+^- nitriltriacetate) affinity column (GE Healthcare) pre-equilibrated with Binding buffer. The column was washed with Binding buffer to elute weakly bound contaminants. The recombinant protein was eluted by passing over the column binding buffer solutions containing increasing imidazole concentrations (50 mM, 100 mM, 150 mM and 250 mM, respectively). The collected eluates were supplemented with a final concentration of 10 mM dithiothreitol (DTT) and tested for purity on SDS gel using precasted gel system (Invitrogen). The pure fractions were incubated overnight with tobacco etch virus protease (Pro-TEV), to remove the hexahistidine tag. After digestion, the protein was concentrated to 2 ml using Millipore concentrators and loaded onto a Superdex 200 300/10 gel filtration column previously equilibrated with 50 mM Tris/HCl, 0.25 M NaCl, 10 mM DTT, pH 7.5 at a flow rate of 1.0 ml/min. 2 ml fractions were collected and the pure protein was identified by SDS PAGE. Protein concentration was determined spectrophotometrically using a molar absorptivity of 49850 M*^−^* ^1^ cm^−1^ at 280 nm based on a molecular mass of 35.190 kDa.

### Temperature dependence of phosphatase activity

The activity assay mixture containing 20 mM Tris-HCl pH 7.5, 200 mM NaCl, 5 mM CaCl_2_, 250 µM DTT, and 100 µM of 6,8-difluoro-4- methylumbelliferyl phosphate (Molecular Probes D6567) in a 0.5 ml final volume was incubated at increasing temperature in a thermostated cuvette. Reaction was started by adding 2–4 µl of purified enzyme at 10°C to 0.5 ml assay mixture equilibrated at the desired temperature. The final enzyme concentration was 1 nM. The solution was thoroughly mixed by pipetting and the fluorescence at 460 nm (360 nm excitation wavelength) was continuously monitored for 360 s in the thermostated cuvette. The fluorescence changes between 150 and 300 s were extrapolated to a standard curve of 6,8-difluoro-7- hydroxy-4-methylcoumarin (Molecular Probes) monitored under the same conditions. The 6,8-difluoro-4- methylumbelliferyl phosphate concentration was well below the *K*_m_ of the wild-type (753 µM) [10] and substrate consumption did not exceed 0.4%, hence the first order rate constant for phosphatase activity, *k* = s^−1^, is obtained from [6,8-difluoro-7- hydroxy-4-methylcoumarin]×s^−1^/[enzyme]. The temperature dependence was fitted nonlinearly to the Arrhenius equation using GraphPad Prism 4.0 to obtain the activation energies (*E*_a_^‡^) for the catalytic reaction
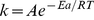
(1)where *k* (s^−1^) is the rate constant at temperature *T* (K), *A* is a reaction specific quantity, *R* the gas constant (1.987 cal×mol^−1^×K^−1^) and *E*_a_ the activation energy of the reaction. Hence, a plot of ln *k* versus 1/*T* gives a straight line with the slope being −*E*_a_/*R*.

### Spectroscopic measurements

Intrinsic fluorescence emission measurements were carried out at 10°C with a LS50B spectrofluorimeter (Perkin-Elmer), at 40.0 µg/ml protein concentration, using a 1.0 cm path length quartz cuvette. Fluorescence emission spectra were recorded from 300–450 nm (1 nm sampling interval), with the excitation wavelength set at 295 nm. Far-UV (190–250 nm) CD spectra were recorded either at a protein concentration of 0.20 mg/ml in a 0.1 cm cuvette or at 40.0 µg/ml in a 0.5 cm cuvette; near-UV (250–320 nm) CD spectra were recorded at protein concentration ranging from 1.0 to 2.4 mg/ml in a 1.0 cm cuvette. Far- and near-UV CD spectra were measured using 0.1, 0.5 and 1.0 cm path length quartz cuvettes and the results obtained were expressed as the mean residue ellipticity [Θ], assuming a mean residue molecular mass of 110 per amino acid residue. All spectroscopic measurements were carried out at 10°C in 20 mM Tris/HCl, pH 7.5 containing 0.2 M NaCl and 200 µM or 2 mM DTT.

### Urea-induced equilibrium unfolding

For equilibrium transition studies, PTPρ wild-type and variants (final concentration 40.0 µg/ml) were incubated at 10°C at increasing concentrations of urea (0–8 M) in 20 mM Tris/HCl, pH 7.5, in the presence of 0.2 M NaCl and 200 µM DTT. After 10 min, equilibrium was reached and intrinsic fluorescence emission and far-UV CD spectra (0.5-cm cuvette) were recorded in parallel at 10°C. To test the reversibility of the unfolding, PTPρ wild-type and variants were unfolded at 10°C in 7.0 M urea at 0.4 mg/ml protein concentration in 25 mM Tris/HCl, pH 7.5, in the presence of 2 mM DTT and 0.2 M NaCl. After 10 min, refolding was started by 10-fold dilution of the unfolding mixture at 10°C into solutions of the same buffer used for unfolding containing decreasing urea concentrations. The final enzyme concentration was 40 µg/ml. After 24 h, intrinsic fluorescence emission and far-UV CD spectra were recorded at 10°C.

### Thermal denaturation experiments

PTPρ variants and wild-type (0.20 mg/ml) were heated from 10°C to 72°C in a 0.1 cm quartz cuvette with a heating rate of 1.0 and 0.5 degree×min^−1^ controlled by a Jasco programmable Peltier element. The dichroic activity at 209 nm and the PMTV were continuously monitored in parallel every 0.5°C [25]. All the thermal scans were corrected for the solvent contribution at the different temperatures. Melting temperature (T_m_) values were calculated by taking the first derivative of the ellipticity at 209 nm with respect to temperature. All denaturation experiments were performed in triplicate.

### Data analysis

The changes in intrinsic fluorescence emission spectra at increasing urea concentrations were quantified as the decrease of relative fluorescence intensity at 345 nm or as the intensity-averaged emission wavelength, 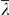, [32] calculated according to
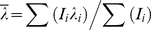
(2)where λ_i_ and *I*_i_ are the emission wavelength and its corresponding fluorescence intensity at that wavelength, respectively. This quantity is an integral measurement, negligibly influenced by the noise, which reflects changes in the shape and position of the emission spectrum. Urea-induced equilibrium unfolding transitions monitored by far-UV CD ellipticities changes was analysed by fitting baseline and transition region data to a two-state linear extrapolation model [33] according to
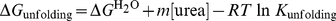
(3)Where Δ*G*_unfolding_ is the free energy change for unfolding for a given denaturant concentration, Δ*G* ^H^_2_^O^ the free energy change for unfolding in the absence of denaturant and *m* a slope term which quantifies the change in Δ*G*_unfolding_ per unit concentration of denaturant, *R* the gas constant, *T* the temperature and *K*_unfolding_ the equilibrium constant for unfolding. The model expresses the signal as a function of denaturant concentration:
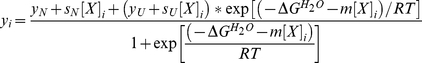
(4)where *y*_i_ is the observed signal, *y*_U_ and y_N_ are the baseline intercepts for unfolded and native protein, *s*_U_ and *s*_N_ are the baseline slopes for the unfolded and native protein, [X]_i_ the denaturant concentration after the ith addition, Δ*G* ^H^_2_^O^ the extrapolated free energy of unfolding in the absence of denaturant, *m* the slope of a Δ*G*_unfolding_ versus [X] plot. Data were globally fitted with the *m* values shared between the data sets; all other parameters were not constrained. The denaturant concentration at the midpoint of the transition, [urea]_0.5_, according to equation 3, is calculated as:
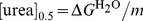
(5)The denaturation curves obtained by plotting the relative fluorescence intensities changes induced by increasing urea concentrations were fitted to the following equation assuming a three-state model:
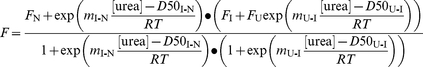
(6)where *F* is the observed fluorescence intensity, *m* is a constant that is proportional to the increase in solvent-accessible surface area between the two states involved in the transition, *D*50_I-N_ and *m*_I-N_ are the midpoint and *m* value for the transition between N and I, respectively, and *D*50_U-I_ and *m*_U-I_ are the midpoint and *m* value for the transition between I and U, respectively [34]. The fluorescence intensity of the intermediate state (I), *F*_I_, is constant whereas that of the folded state (N) and of the unfolded state (U), *F*_N_ and *F*_U_, respectively, has a linear dependence on denaturant concentration
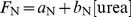
(7)

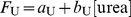
(8)where *a*_N_ and *a*_U_ are the baseline intercepts for N and U, *b*_N_ and *b*_U_ are the baseline slopes for N and U, respectively. All unfolding transition data were fitted by using Graphpad Prism 4.0.

Far-UV CD spectra recorded as a function of urea concentration were analyzed by SVD using the software MATLAB (Math-Works, South Natick, MA) to remove the high frequency noise and the low frequency random errors and to determine the number of independent components in any given set of spectra. CD spectra in the 213–250 nm or in the 250–320 nm region were placed in a rectangular matrix *A* of *n* colu mns, one column for each spectrum collected at each time. The *A* matrix is decomposed by SVD into the product of three matrices: *A* = U*S*V ^T^, where *U* and *V* are orthogonal matrices and *S* is a diagonal matrix. The *U* matrix columns contain the basis spectra and the *V* matrix columns contain the urea dependence of each basis spectrum. Both *U* and *V* columns are arranged in terms of decreasing order of the relative weight of information, as indicated by the magnitude of the singular values in *S*. The diagonal *S* matrix contains the singular values that quantify the relative importance of each vector in *U* and *V*. The signal-to-noise ratio is very high in the earliest columns of *U* and *V* while the random noise is mainly accumulated in the latest *U* and *V* columns. The wavelength averaged spectral changes induced by increasing denaturant concentrations are represented by the columns of matrix *V*; hence, the plot of the columns of *V* versus the denaturant concentrations provides information about the observed transition.
